# Fractography of white charcoal reveals past fungal infection and embolism in the secondary xylem via SEM

**DOI:** 10.1186/s42649-026-00126-w

**Published:** 2026-03-03

**Authors:** Chul Jong Yoon, Ki Woo Kim

**Affiliations:** 1 Department of Pathology, Seoul National University Hospital, Seoul, 03080 Republic of Korea; 2 Department of Forest Ecology and Protection, Kyungpook National University, Sangju, 37224 Republic of Korea; 3 Tree Diagnostic Center, Kyungpook National University, Sangju, 37224 Republic of Korea; 4 Present address: Research Institute of Electron Microscopy, Gimpo, 10066 Republic of Korea

**Keywords:** Carbonized wood, Oak, Pit, *Raffaelea*, Tylosis, Vessel

## Abstract

The internal structures of fractured white charcoal were investigated using a scanning electron microscope. The charcoal was fractured using a razor blade and hammer, gold-coated, and observed under the electron microscope. Both embolized and conductive vessels coexisted across the transverse surfaces of the fractured charcoal. Vessels were predominantly ellipsoidal, with an average diameter of approximately 250 μm. Embolized vessels exhibited membranous tyloses within their lumens. The presence of ring-porous wood, axial parenchyma cells, and xylem fibers within the secondary xylem implies that the white charcoal was produced from a *Quercus* species. Fungal hyphae were observed to proliferate, branch, and sporulate on the secondary cell walls of vessels. The conidiogenous cells and conidia closely resembled those of a fungal pathogen known to cause oak wilt disease in South Korea. Fungal hyphae were also found within the pits of the secondary cell walls. These observations suggest that the oak tree used for charcoal production in this study may have been diseased. These results indicate that wood structures are preserved through the white charcoal production process, allowing the observation of fungal structures within the host.

## Introduction

Before the emergence of modern fossil fuels and electricity, charcoal was the primary fuel source due to its relatively high calorific value, availability, and transportability (Krebs et al. 2017). While charcoal is mainly produced from wood, biochar can be derived from a wide variety of biomass sources, including plant residues, such as peanut shells, corn straw, and bamboo, as well as animal wastes, such as cattle, pig, and chicken manure (Wang et al. 2024). Charcoal is classified into three categories based on the carbonization process producing it: (i) black charcoal, (ii) activated charcoal, and (iii) white charcoal (Pijarn et al. 2021). These charcoals are produced at temperatures below 400 °C, from 400 to 800 °C, and above 1,000 °C, respectively (Pijarn et al. 2021). More specifically, white charcoal is produced by pyrolyzing the wood at ~ 200–400 °C for several days, and the temperature is increased to above 1,000 °C toward the end of the process (Chia et al. 2014; Miura 1931).

The term “white charcoal” originates from the practice of quenching the wood with ash, which imparts a pale grey hue to its surface (Chia et al. 2014). In South Korea, 90% of all white charcoal is produced in Gangwon Province from oak trees, primarily Chinese cork oak (*Quercus variabilis*), and is utilized for crafts, water treatment, and various other daily applications (Kwon et al. 2012). As electrical resistivity decreases with increasing carbonization temperature, likely due to the formation of ultrafine graphite-like crystallites, accompanied by the appearance of sodium compound crystals (Xiao et al. 2012), white charcoal exhibits electrical conductivity, rendering it a suitable electrode material for fuel cells or batteries (Kwon et al. 2013).

The morphological characteristics of white charcoal have been examined several times using scanning electron microscopy (SEM) and transmission electron microscopy (TEM). Chia et al. (2014) analyzed the morphologies and elemental compositions of white charcoal particles using SEM, TEM, and energy-dispersive X-ray spectroscopy. Pijarn et al. (2021) examined the microstructural features of bamboo xylem in white charcoal via SEM. Additionally, morphological changes in the xylem structure of *Q. variabilis* were compared across carbonization temperatures using SEM, after samples were split and sputter-coated with gold–palladium (Kim and Hanna 2006). SEM was employed to evaluate the impact of different kiln types on the anatomical structures of white charcoal (Kwon et al. 2018).

However, limited information is available regarding the cleaving or fracturing methods used to reveal the internal anatomical structure of white charcoal for SEM observation. There is a scarcity of visual evidence regarding microbial behavior within the internal structure of white charcoal. The objective of this study was to assess one such cleaving technique for use in the characterization of microbial behavior within the charcoal matrix via SEM.

## Materials and methods

### White charcoal

Three samples of commercial white charcoal (approximately 15 cm in diameter) were obtained from a wood artisan in Korea. The samples, presumed to have been made from oak species in 2005, were purchased by the artisan for use in creating decorative wood crafts. The charcoal lumps appeared white to pale gray overall and produced a metallic sound when two collided.

### Fracturing under ambient conditions

A total of three air-dried white charcoal samples were selected for electron microscopy. At ambient temperature, a razor blade was inserted as a makeshift chisel into each charcoal lump and then hit with a hammer to split the charcoal lump into smaller pieces. The ability of this technique to reveal internal surfaces with no need for a polishing or smoothing procedure was then evaluated.

### Scanning electron microscopy

Approximately 1 × 1 × 1 cm fractured charcoal pieces were mounted on an aluminum stub with double-sided copper tape without any other conventional preparation techniques, such as chemical fixation, ethanol dehydration, or critical point drying, applied. The mounted pieces were coated with gold using an ion coater (IB-3; Eiko, Tokyo, Japan). The specimens were then observed using a scanning electron microscope (S-520; Hitachi, Tokyo, Japan) operated at 20 kV. Morphological measurements of vessels and pits were performed, and the means and standard deviations (SD) were calculated using a software (Microsoft Excel; Microsoft Corp., Redmond, WA, USA).

## Results

**Fig. 1:**
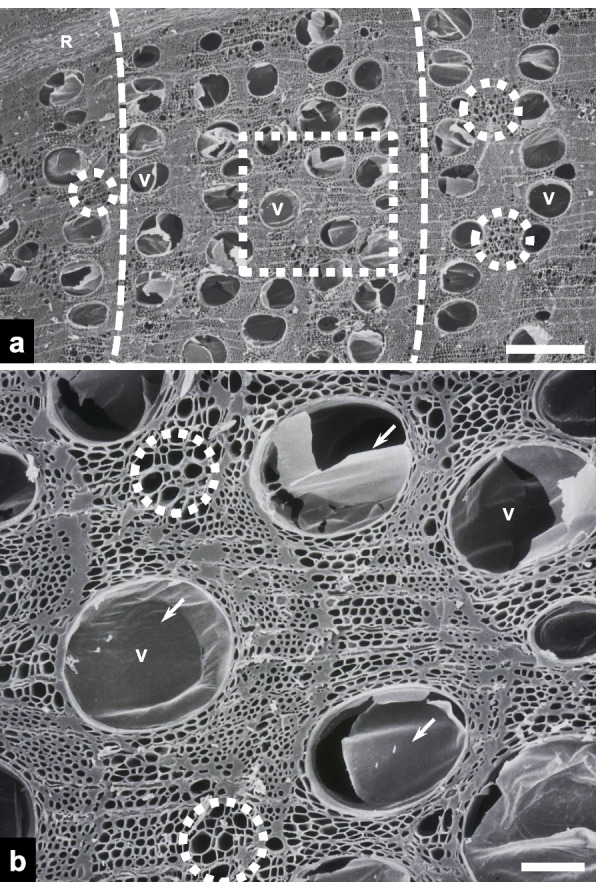
Scanning electron micrographs of transverse surfaces of heartwood in the fractured charcoal. **a** An overview of the heartwood (scale bar = 500 μm). Note that most vessels (Vs) are embolized. Dotted circles highlight clusters of axial parenchyma cells. Dotted line = earlywood. R = ray. **b** A higher-magnification view of the dotted rectangle in panel **a** (scale bar = 100 μm). Note the tyloses (arrows) in the embolized Vs and axial parenchyma cells in dotted circles

Scanning electron microscopy revealed the transverse surface of the heartwood in charcoal fractured via the razor blade-and-hammer method (Fig. 1a). Vessels were primarily concentrated in the earlywood and nearly all were embolized. They were predominantly ellipsoidal, with a mean diameter of 252.82 ± 27.09 μm (*n* = 20, mean ± SD; Fig. 1b). Embolized vessels exhibited ruptured tyloses within their lumen. Clusters of axial parenchyma cells were observed between the larger vessels.

**Fig. 2:**
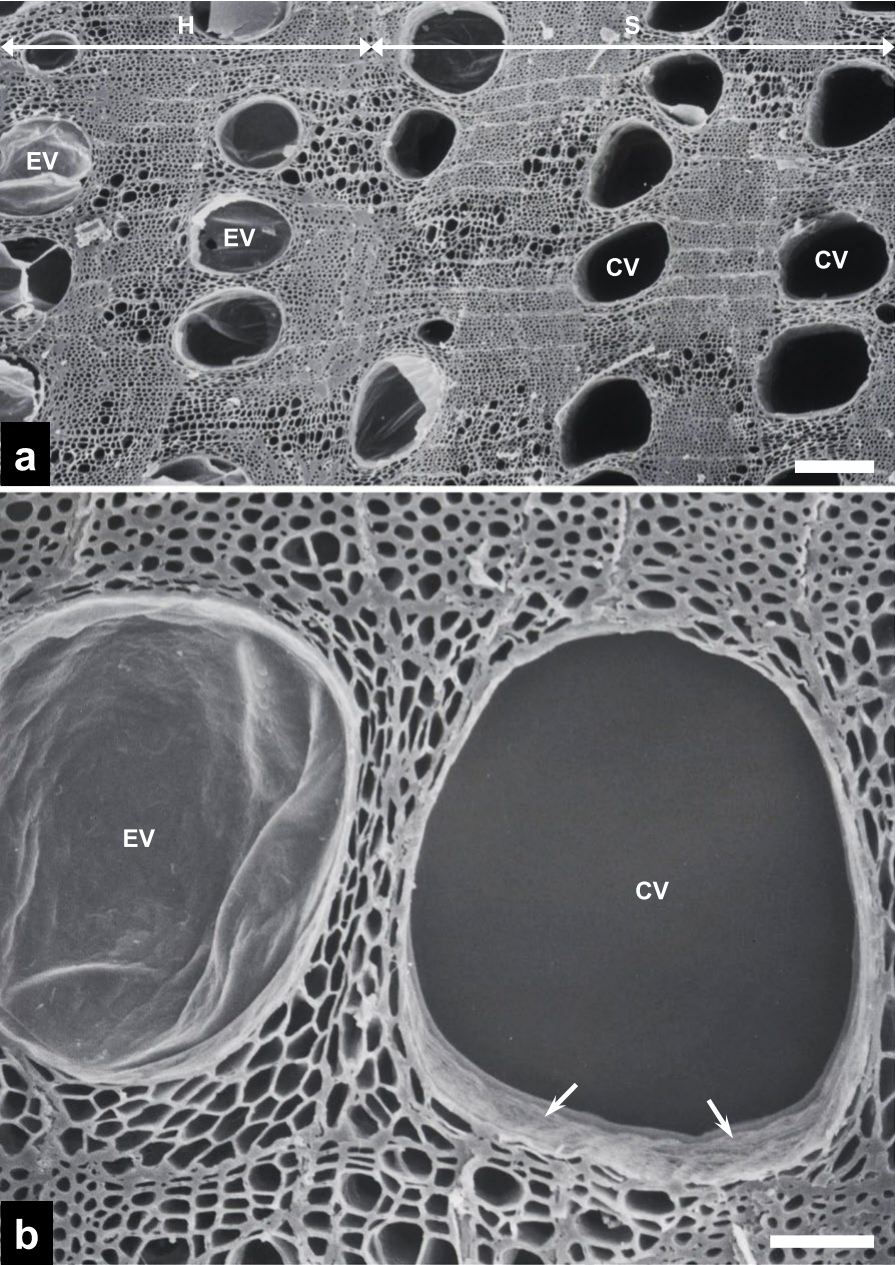
Scanning electron micrographs of transverse surfaces of the transition zone in the fractured charcoal. **a** An overview of the transition zone between the heartwood (H) and sapwood (S) (scale bar = 200 μm). Note the presence of both embolized vessels (EVs) containing tyloses and conductive vessels (CVs) with open lumens. **b** A higher-magnification view of an EV and a CV (scale bar = 50 μm). Note the corrugated secondary cell walls (arrows) within the CV

Embolized and unoccluded vessels were observed to coexist in an approximately 1:1 ratio across the transverse fracture surface within the transition zone (Fig. 2a). While vessels in the heartwood were embolized with tyloses, those in the sapwood were conductive, with open lumens. Magnified views revealed the membranous, bag-like appearance of the tyloses (Fig. 2b). Corrugated secondary cell wall surfaces were clearly discernible within the conductive vessels.

**Fig. 3:**
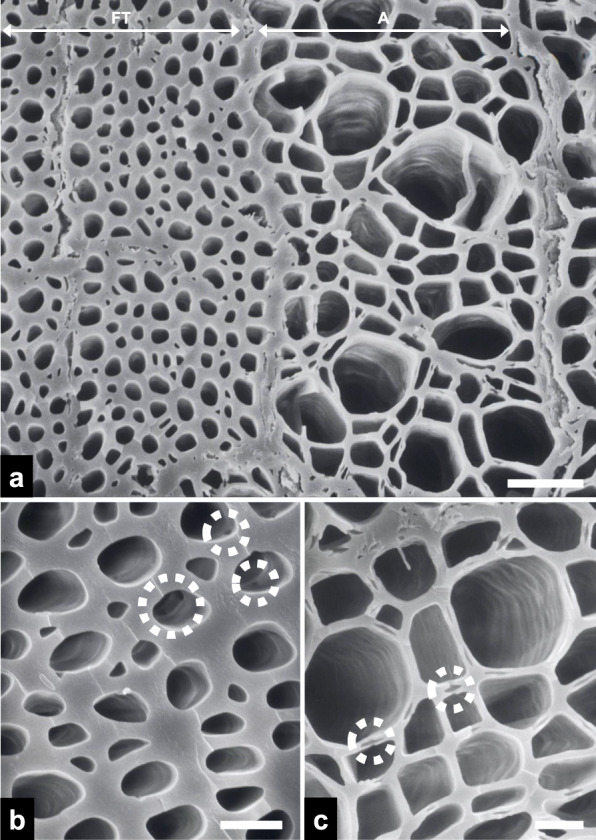
Scanning electron micrographs of transverse surfaces in the fractured charcoal. **a** An overview image including the fiber-tracheid (FT) and axial parenchyma cell (A) regions (scale bar = 30 μm). Note the variation in cell wall thickness between the two xylem components. **b** A higher-magnification view of FTs, with pits (dotted circles; scale bar = 5 μm). **c** A higher-magnification view of As, with cell–cell junctions (dotted circles; scale bar = 10 μm)

Clusters of xylem components with varying diameters—comprising fiber-tracheids and axial parenchyma cells—were observed on the transverse surface of the fractured charcoal (Fig. 3a). Fiber-tracheids exhibited narrower diameters (~ 5 μm) and thicker cell walls (~ 2 μm) than adjacent axial parenchyma cells (Fig. 3b), and the inner surfaces of pits were discernible within the fiber-tracheids. Magnified views revealed the cell–cell junctions between adjacent axial parenchyma cells (Fig. 3c).

**Fig. 4:**
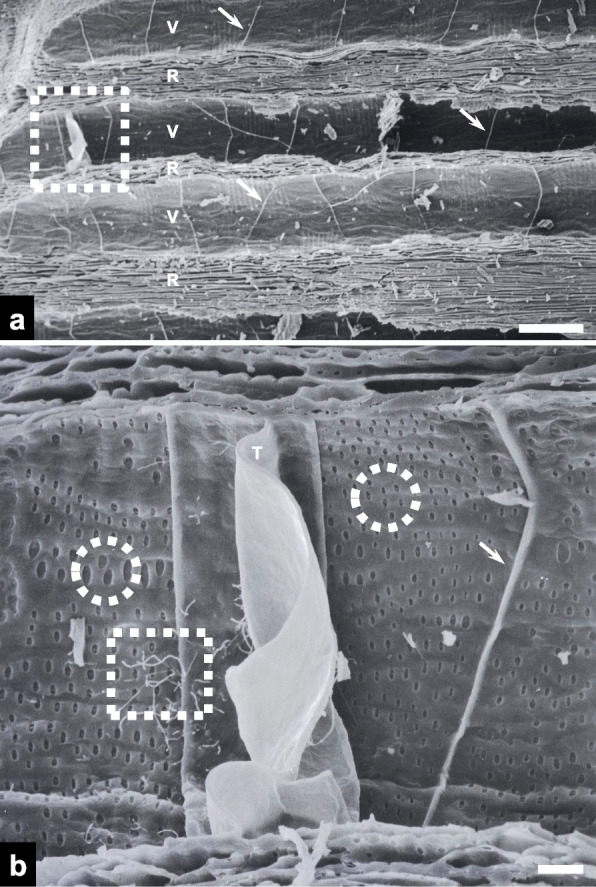
Scanning electron micrographs of radial surfaces in the fractured charcoal. **a** An overview image including vessels (Vs) and rays (Rs) (scale bar = 200 μm). Note the simple perforation plates (arrows). **b** A higher-magnification view of the dotted rectangle in panel **a** (scale bar = 20 μm). Note the fungal hyphae (dotted rectangle), embolizing membranous tylose (T) adjacent to the fungal hyphae, and oval-shaped pits (dotted arrows)

Vessels and rays were clearly visible on the radial fracture surfaces of the charcoal (Fig. 4a). There were several simple perforation plates within the vessels. Magnified views revealed fungal hyphae adjacent to a membranous tylose within a vessel (Fig. 4b). Hyphae were not observed to pass through the tylose. Vessel pits of varying shapes were found on the secondary cell walls of the vessels.

**Fig. 5:**
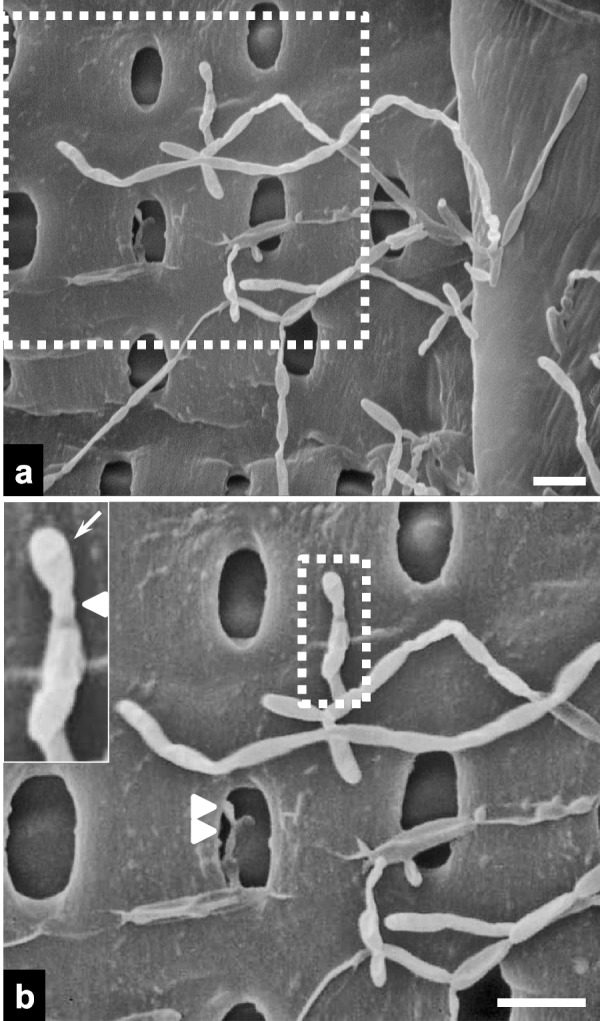
Scanning electron micrographs of fungal hyphae on radial vessel surfaces in the fractured charcoal. **a** A higher-magnification view of the dotted rectangle in Fig. 4b. **b** A higher-magnification view of the dotted rectangle in panel **a**. The inset shows a higher-magnification view of the conidiogenous cell in the dotted rectangle in panel **b**. Note the obovoid conidium (arrow), protruding base (arrowhead), and hyphal growth in a vessel pit (double arrows; scale bars = 5 μm)

Fungal hyphae proliferated, branched, and sporulated on the secondary cell walls of the vessels (Fig. 5a). A magnified view revealed a conidiogenous cell with a flat scar or annellation (Fig. 5b). The conidium was oval-shaped and measured approximately 3 μm in length. Fungal hyphae were also found within the pits of secondary cell walls.

**Fig. 6:**
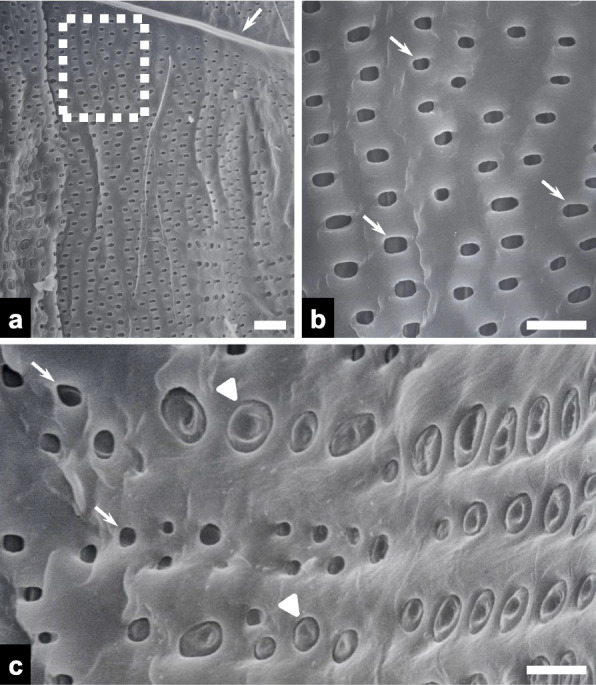
Scanning electron micrographs of radial vessel surfaces in the fractured charcoal. **a** Overview image showing vessel pits (scale bar = 20 μm). Note the simple perforation plates (arrow). **b** A higher-magnification view of the vessel pits (arrows) in the dotted rectangle in panel **a** (scale bar = 10 μm). **c** A higher-magnification image of conductive vessel pits (arrows) and embolized vessel pits filled with ray cells (arrowheads; scale bar = 10 μm)

The secondary cell walls of the vessels exhibited a high density of oval-shaped vessel pits (Fig. 6a), and higher magnification views revealed vessel pits with an average length of 3.14 ± 0.5 μm (*n* = 20; Fig. 6b). Both open and embolized vessel pits filled with ray cells of varying diameters were also observed on the secondary cell walls of the vessels (Fig. 6c).

## Discussion

This study described the anatomical structures of wood in fractured white charcoal—including vessels, axial parenchyma cells, pits, and tyloses—using SEM. Fracturing specimens under ambient conditions before observation effectively revealed well-preserved secondary xylem structures, which survived the high-temperature processing required for white charcoal production. The presence of ring-porous wood, axial parenchyma cells, and xylem fibers within the secondary xylem suggests that the white charcoal was produced from *Quercus* species (Eom 2015). This identification is supported by the fact that most white charcoal in South Korea is manufactured from the wood of this genus (Kwon et al. 2012). Although the white charcoal was not stained for histological analysis, its anatomical structures could be partially identified in SEM images based on cell morphology and wall thickness.

Tyloses-containing embolized vessels were prevalent in the heartwood sections of the fractured white charcoal. First described in the late 1680 s, “tylosis” is derived from the Greek term meaning “bag” or “container” (De Micco et al. 2016). They are ingrowths of parenchyma cells passing through the pits into the lumens of xylem vessels (Leśniewska et al. 2017). While some tyloses retained near-intact membranous morphologies, the majority became ruptured during the production process. Tyloses appeared to serve as a physical barrier, deterring the spread of fungal hyphae within the vessels, as evidenced by the absence of hyphae passing through them (Fig. 4b). The membranous, bag-like appearance of the tyloses in this study is consistent with SEM observations in other plant species, such as *Vitis* species infected with *Xylella fastidiosa* (Fritschi et al. 2008) and *Morus australis* (De Micco et al. 2016). Xylem embolism has been recorded in a diverse range of oak species, including white oak (*Q. alba*), using fluorescence microscopy (Kitin et al. 2021); California black oak (*Q. kelloggii*), using X-ray micro-computed tomography; and *Q. kingiana*, using bright-field microscopy (Gupta and Gupta 2020; Percolla et al. 2021). These observations corroborate the results obtained in this study.

The presence of ray cells within the pits of embolized vessels likely marks the initial stage of tylosis budding. Subsequent budding and enlargement may then lead to the formation of a complete tylosis that fully occludes the vessels (Ishida and Ohtani 1968). Tylosis buds were also identified within the wood structure of *Q. variabilis* charcoal (Kim and Hanna 2006) and *Eucalyptus globulus* chips (Leitch et al. 1999). The mechanisms by which nascent tylosis buds merge and enlarge within the vessels remain to be fully elucidated in oak species.

An unexpected finding was the observation of fungal hyphae within vessels. The fungal hyphae within the vessels exhibited morphological features highly consistent with *Raffaelea quercus-mongolicae*, a pathogen known to cause oak wilt disease in South Korea (Kim et al. 2009). This species is characterized by conidiogenous cells with flat scars and obovoid to pyriform conidia, measuring approximately 4–10 μm in length, as previously described (Kim et al. 2009). Several species of *Raffaelea* have also been associated with oak decline in other countries (Inácio et al. 2021; Simmons et al. 2016). In addition, vessel occlusion caused by tylosis formation in response to the fungal pathogen infection has been observed in infected oak trees (Jeon et al. 2020). Oak wilt disease was first reported in Korea in 2004, and it occurs most frequently in Mongolian oak (*Q. mongolica*) (Kim et al. 2009). The number of trees in Korea killed annually by this disease peaked at approximately 331,000 in 2011 before declining to about 157,000 in 2019 (Choi et al. 2022). Given that the wood was harvested in 2005, we hypothesized that the trees used to produce the white charcoal had been infected by the pathogenic fungus in the stand, triggering tylosis as a defensive response. As oak trees displaying wilt symptoms in the field may be harvested for use as construction timber, mushroom cultivation substrate, or charcoal production, the SEM observations imply that the oak tree used to produce the charcoal in this study may have been diseased. Further investigation is necessary to track the production history of white charcoal and thereby establish a chronological foundation for dendrochronology in Korea.

The presence of fungal hyphae within the white charcoal suggests that the fungi had already colonized the wood prior to the carbonization process. Meanwhile, post-production colonization by saprophytes, wood-decay fungi, or even charcoal-degrading fungi can frequently occur on the surface of white charcoal. However, fungal invasion after charcoal production is unlikely to occur, as the occluded vessels and tyloses act as a physical barrier. Consequently, it is reasonable to deduce that fungal colonization occurred prior to carbonization, retaining distinct morphological details, as suggested previously (Moskal-del Hoyo et al. 2010).

Notably, fungal hyphae were localized within the vessel pits of the secondary cell walls (Fig. 5b), and the vessel pit apertures were large enough to permit the passage and lateral migration of fungal hyphae. Such fungal behavior suggests that this is a mechanism for hyphal colonization from infected vessels into adjacent vessels, facilitating spread within the host. Hyphal entrance into bordered vessel pits in *Picea abies* heartwood was observed after 6 weeks of incubation with the white rot fungus *Physisporinus vitreus* (Schwarze et al. 2006). Based on their anatomically distinct growth rings and ability to grow under a wide ecological range, oak species have commonly been employed for dendrochronological analyses (Haneca et al. 2009). Taken together, our results, along with these earlier findings, suggest that oak charcoal could be utilized as a proxy for inferring historical pathological events in the field. As these pathological inferences relied solely on morphological fungal identification, DNA sequence-based studies are essential to validate host-parasite interactions and provide more robust evidence for these past pathological occurrences.

Other anatomical wood structures noted in this study include perforation plates, fiber-tracheids, and axial parenchyma cells. The perforation plates within the vessels were simple in the white charcoal, resembling those seen in three other oak species (Olfat and Pourtahmasi 2010). Both pits and expanded pit chambers have been shown in stained cross sections of fiber-tracheids in *Paracryphia* species (Olson 2023). These observations confirm that the structural integrity of wood can be retained through the white charcoal production process.

## Conclusions

The razor blade-and-hammer fracturing method employed to expose the internal structure of white charcoal proved to be effective for visualizing wood anatomy. The tylosis-induced embolisms and fungal behavior observed in the wood suggest that oak charcoal can potentially be used as a dendrochronological tool for inferring past forest disease outbreaks. Combined with state-of-the-art imaging modalities, this simple and rapid fracturing method can provide novel insights into plant–microbe interactions in both past and present ecosystems.

## Data Availability

Data and materials will be made available upon request.

## Abbreviations

SEM: Scanning electron microscopy
TEM: Transmission electron microscopy
